# A perspective on auxetic nanomaterials

**DOI:** 10.1186/s40580-017-0104-3

**Published:** 2017-04-16

**Authors:** Harold S. Park, Sung Youb Kim

**Affiliations:** 1 Department of Mechanical Engineering, Boston University, Boston, MA 02215 USA; 2 Department of Mechanical Engineering, Ulsan National Institute of Science and Technology, Ulsan, 44919 South Korea

**Keywords:** 2D materials, Auxetic, Negative Poisson’s ratio

## Abstract

Nanomaterials have recently been found to exhibit auxetic behavior, or a negative Poisson’s ratio, whereby the lateral dimensions of the material expand, rather than shrink, in response to applied tensile loading. In this brief review, we use the form of question–answer to highlight key points and outstanding issues related to the field of auxetic nanomaterials.

## What are auxetic materials and why are they of interest?

Most materials, when subject to tensile mechanical forces, elongate in the direction of the applied force, and contract in the directions normal to the applied force. This phenomenon is described mathematically by the Poisson’s ratio $\nu$. In two-dimensions, if a material is stretched in the *x*-direction, it would contract in the *y*-direction, and its Poisson’s ratio would be defined as $\nu _{xy}=-\epsilon _{y}/\epsilon _{x}$, where $\epsilon$ is the strain in either the *x*- or *y*-directions. The Poisson’s ratio is typically positive because the strain normal to the applied load $\epsilon _{y}$, is usually negative. Classical elasticity theory does allow for a negative Poisson’s ratio (NPR), and the Poisson’s ratio for isotropic materials can lie in the range $-1< \nu < 0.5$.

Materials that expand in the lateral directions in response to an applied tensile force were termed auxetic by Evans [1]. Beginning with seminal experiments of Lakes, who demonstrated NPR in a foam structure [2], various structures and materials have been shown to exhibit the NPR phenomenon [3–15].

Auxetic materials have been widely studied for a range of target applications due to this unique mechanical property. Auxetic materials also may have enhanced shear resistance, indentation resistance, fracture toughness, and enhanced damping of wave propagation and vibration transmission in periodic auxetic structures [16, 17].

## What mechanisms for auxetic behavior in bulk materials have been observed to hold in nanomaterials?

One of the first mechanisms for enabling auxetic behavior in bulk materials was developed by Lakes, in which a structural foam was engineering to exhibit auxetic behavior via a microstructure containing a re-entrant unit cell with two orthogonal hinges. When this structure is under mechanical tension, one of the hinges opens along the tensile axis, whereas the other hinge expands in the lateral direction, resulting in a negative Poisson’s ratio in the out-of-plane direction [2].

**Fig. 1:**
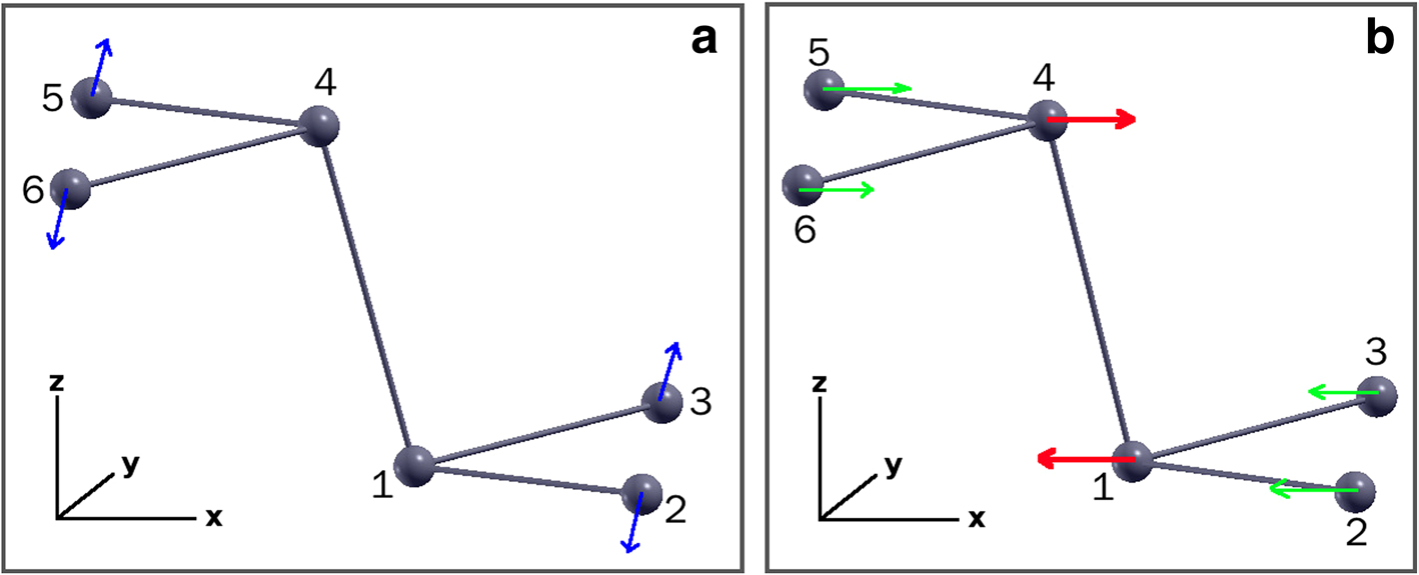
The evolution of local structure in single-layer BP during uniaxial tension in the y-direction.** a** BP is stretched in the y-direction, i.e. atoms are moved in the direction of the attached* arrows* (*blue online*).** b** To accommodate the tension in the y-direction, BP contracts in the x-direction, i.e. atoms 1 and 4 move inward along the attached* arrows* (*red online*). The 1–4 bond thus becomes more closely aligned with the vertical (z)-direction. The* green arrows* display the movement of the four surrounding atoms following the movement of atoms 1 and 4 (Reproduced with permission from Jiang and Park [18]. Copyright 2014 Macmillan Publishers Ltd.)

Interestingly, certain two-dimensional (2D) nanomaterials have an intrinsic crystal structure that precisely mimics the re-entrant hinge mechanism first utilized by Lakes. One example is black phosphorus, which has, as shown in Fig. 1, a puckered lattice structure [18], with the two hinges formed by the angles $\theta _{546}$ and $\theta _{214}$, thus demonstrating a nanoscale version of the re-entrant hinge mechanism.

Black phosphorus is not the only puckered two-dimensional material. Other such materials include orthorhombic arsenic [19], borophane [20], and also the monochalcogenides, like GeSe and SnS [21].

**Fig. 2:**
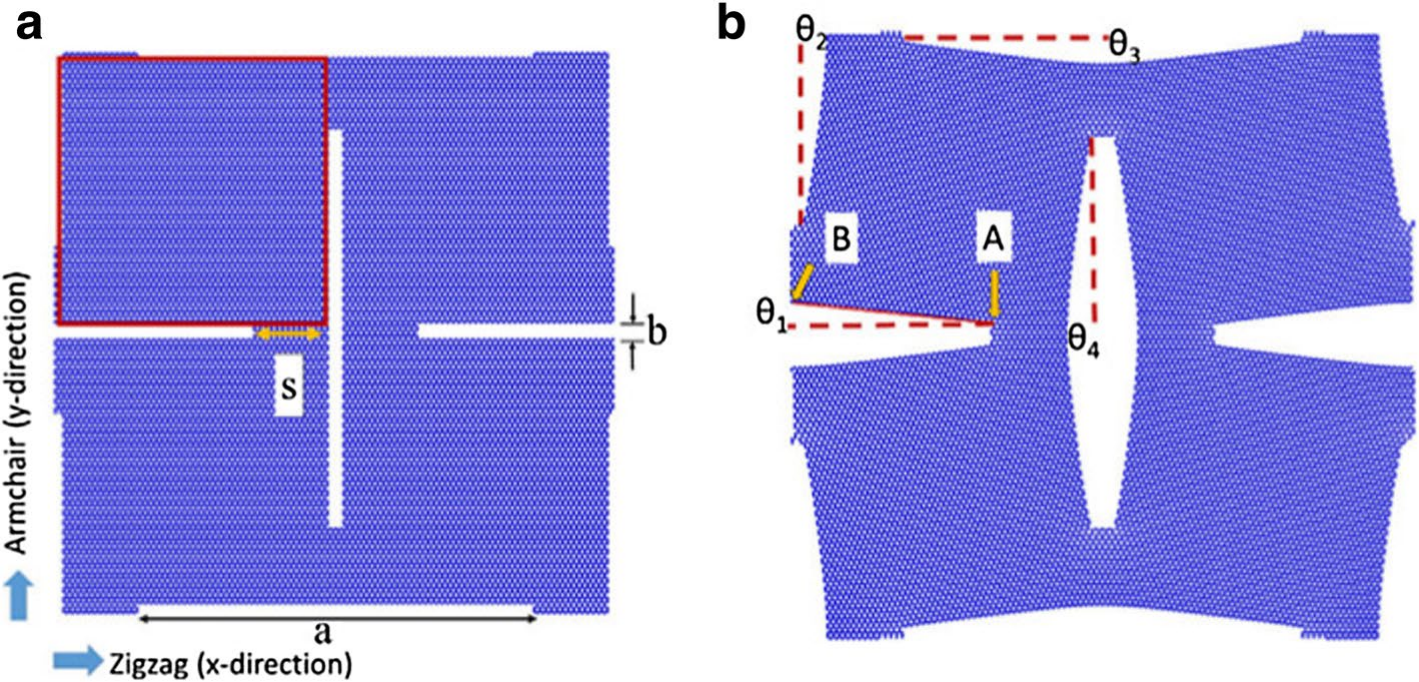
Patterned porous graphene under uniaxial loading. Size = 24.6 × 24.7 nm^2^. Configurations of the patterned porous graphene at strain** a** $\epsilon =0$ and** b** $\epsilon =0.1$ under uniaxial loading in the zigzag direction (Reproduced with permission from Ho et al. [43]. Copyright 2016, Wiley-VCH)

Bulk materials have also been successfully patterned through cutting techniques to induce NPR [8, 22–25], and this approach also has produced similar success in creating NPR nanomaterials. Figure 2 illustrates this for patterned graphene, which exhibits NPR because the internal units rotate in response to the applied strain.

## What new mechanisms for auxetic behavior emerge at the nanoscale?

**Fig. 3:**
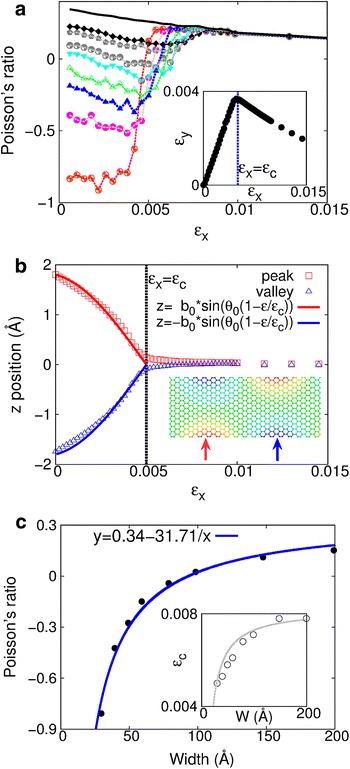
Poisson’s ratio for graphene from Set I.** a** Strain dependence for Poisson’s ratio. Inset displays the strain–strain relation for graphene with width 29.51 Å, in which graphene expands in the y-direction when it is stretched in the x-direction by strain smaller than $\epsilon _c=0.005$, indicating the NPR effect.** b** The strain dependence for the z positions of two atoms in the peak (valley) of the warped edge in graphene. Data are fitted to functions $z=\pm b_0\sin [\theta _0(1-\epsilon /\epsilon _c)]$ with constraint $b_0=z_0/\sin \theta _0$.* Inset* displays these two atoms; i.e. the atom on the peak (*red arrow*) and valley (*blue arrow*) of the warped edge. Both atoms fall into the graphene plane for strain larger than $\epsilon _c$.** c** Width dependence for the Poisson’s ratio. * Inset* shows the critical strain in graphene ribbons of different width (Reprinted (adapted) with permission from Jiang and Park [28]. Copyright (2016) American Chemical Society)

One of the distinct nanoscale effects that strongly impacts the mechanical properties of nanomaterials is the surface stress (or edge stress for 2D materials) [26, 27], which result from the fact that surface atoms have fewer bonding neighbors than atoms that lie within the material bulk. Edge buckling due to edge stresses has been found to induce NPR in narrow graphene ribbons [28], as illustrated in Fig. 3. Similarly, surface stresses have previously been shown to induce phase transformations [29], as well as shape memory and pseudoelasticity in metal nanostructures [30, 31], and were recently shown to, by inducing phase transformations in metal nanoplates, induce auxetic behavior in the metal nanostructures, including nanoplates [32, 33] and nanowires [34].

2D nanomaterials, due to being only a single, or few atoms thick, are mechanically resistant to in-plane stretching, but can easily be induced to deform out of the 2D plane due to their small bending modulus [35, 36]. Thus, one mechanism that has been widely exploited to generate NPR in 2D materials, is that of rippling. Rippling induces NPR in 2D materials because in-plane stretching causes the ripples to flatten, and an expansion of the in-plane dimensions, resulting in a NPR.

**Fig. 4:**
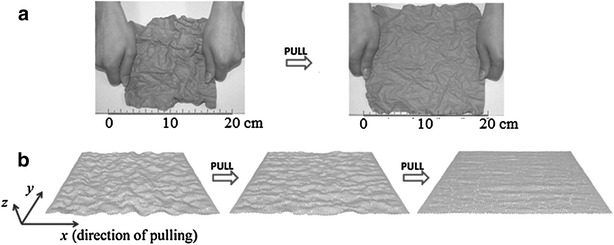
**a** Typical images of a crumpled sheet of paper at different levels of applied strain and** b** typical images of a graphene sheet having 3.0% defects at different levels of applied strain. Note that both systems undergo a de-wrinkling process so as to assume a more planar conformation resulting in an in-plane negative Poisson’s ratio (Reproduced with permission from Grima et al. [38]. Copyright 2016 Wiley-VCH)

Rippling-induced NPR has been generated in 2D materials in various ways, including thermally-induced ripples [37], introduction of 5-8-5 double vacancies in graphene [38] and hydrogenation of graphene [39]. The mechanism by which rippling induced by vacancies in graphene leads to NPR is illustrated in Fig. 4.

## What are the key challenges and open issues?

There are many possibilities and challenges in the field of auxetic nanomaterials, particularly since the first report of NPR in a single-crystal nanomaterial occurred only in 2014 for both black phosphorus and metal nanoplates. One key challenge is in determining how an *in-plane* NPR can be induced in nanomaterials. This is because currently, essentially all examples of NPR reported so far for nanomaterials are *out-of-plane*, i.e. via the rippling mechanism, and also the hinge mechanism for black phosphorus. However, very few reports of in-plane NPR have emerged. For example, transition metal dichalcogenides MX_2_ (MoSe_2_, MoTe_2_, WSe_2_, WTe_2_, TcTe_2_, ReSe_2_, and ReTe_2_) shows in-plane auxeticity in the elastic range [40], and pristine graphene [41] and semi-fluorinated graphene [42] exhibit in-plane auxeticity for large tensile strains exceeding about 6 and 9%, respectively.

Another question that emerges is to move past the discovery phase of NPR in nanomaterials, and to develop a mechanistic understanding of how to controllably tune the NPR in nanomaterials, either by controllably altering the structure of nanomaterials, most likely via cutting and patterning.

Finally, most reports of NPR have so-far been in 2D materials. However, the mechanisms for NPR in 2D materials, which to-date have primarily exploited the low bending modulus to achieve an out-of-plane NPR, may not be operant for other nanomaterials, like 1D nanowires. Thus, it remains to be determined what new mechanisms can be found for other important nanomaterials.
